# Supplementary material for: The genomic and transcriptomic landscape of advanced renal cell cancer for individualized treatment strategies
Source: Sci Rep. 2023 Jul 3;13:10720. doi: 10.1038/s41598-023-37764-z (PMC10318030; doi:10.1038/s41598-023-37764-z)
Supplement: Supplementary file 1 — Supplementary Information 1. [file 41598_2023_37764_MOESM1_ESM.pdf]

Supplementary Figure 1

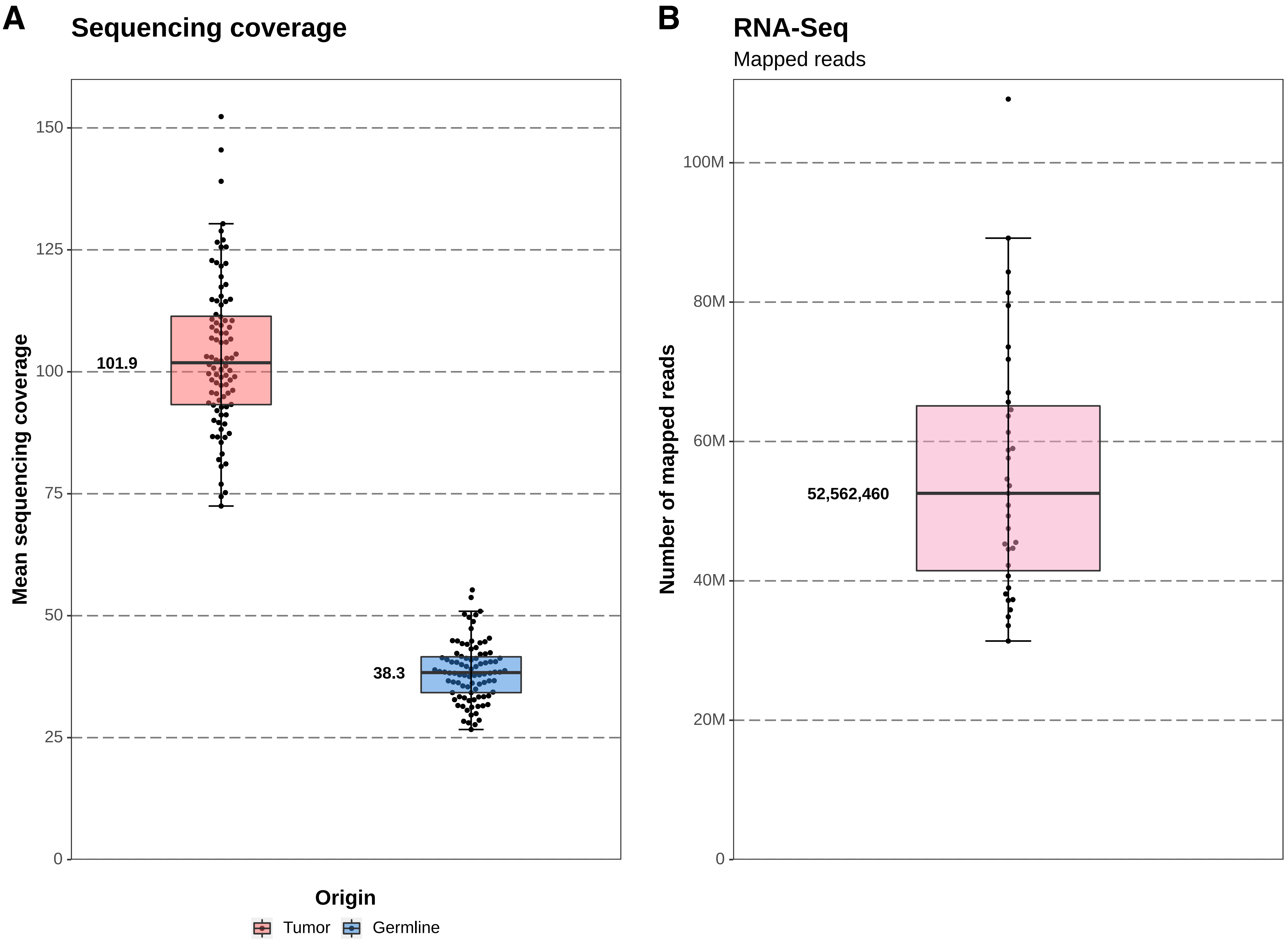

Supplementary figure 1: Whole-genome sequencing and RNA-Seq metrics
Panel A shows the genome-wide mean sequencing coverage for each sample (N=91) as boxplots, for both tumor (red) and whole-blood (blue). Panel B shows the number of mapped reads for the RNA-Seq samples (N=28).
