# Supplementary material for: The genomic and transcriptomic landscape of advanced renal cell cancer for individualized treatment strategies
Source: Sci Rep. 2023 Jul 3;13:10720. doi: 10.1038/s41598-023-37764-z (PMC10318030; doi:10.1038/s41598-023-37764-z)
Supplement: Supplementary file 2 — Supplementary Information 2. [file 41598_2023_37764_MOESM2_ESM.pdf]

Supplementary figure 2

Overview of Mutational Frequencies

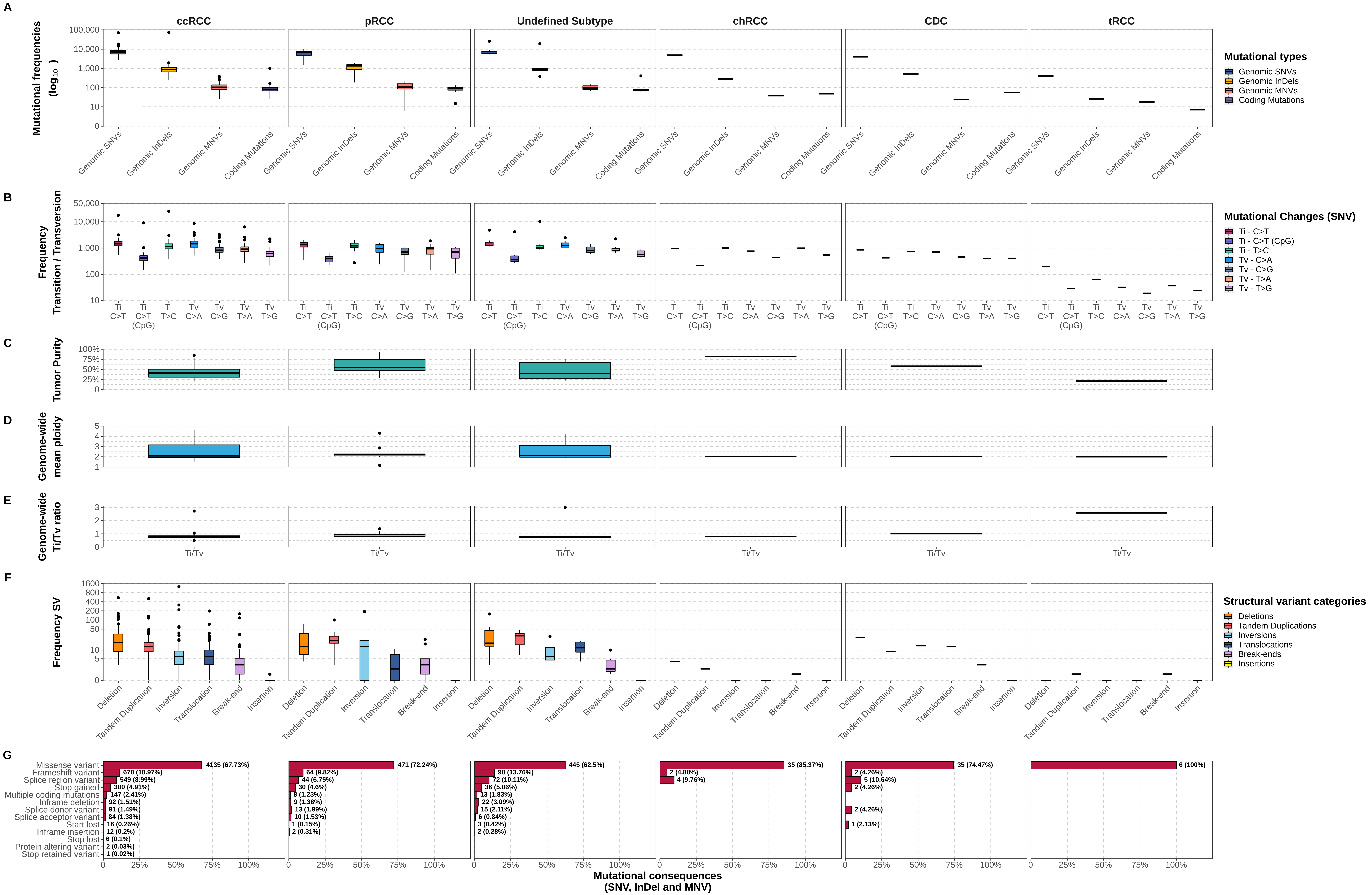

Supplementary figure 2: Mutational overview of the whole-genome sequenced advanced Renal Cell Carcionoma (RCC) cohort (N = 91)

Track A shows the genome-wide and coding region frequency for three small mutational types (single nucleotide variant (SNV), multi nucleotide variant (MNV) and InDel). Track B shows the frequency of each type of transition and transversion including C to T in CpG context. Tracks C, D and E show boxplots of the tumor purity, the estimated genome-wide ploidy for the tumors and the ratio of transitions (Ti) to transversions (Tv). Track F shows the frequency in boxplots of six types of structural variants (translocation, deletion, tandem duplication, break-ends, insertion and inversion), and track G shows the consequences at coding level of the small mutational types. ccRCC = clear cell renal cell carcinoma. pRCC = papillary renal cell carcinoma. Undefined subtype = renal cell carcinoma, with undefined subtype. chRCC = chromophobe renal cell carcinoma. CDC = collecting duct carcinoma. tRCC = tubulocystic renal cell carcinoma.
