# Supplementary material for: The genomic and transcriptomic landscape of advanced renal cell cancer for individualized treatment strategies
Source: Sci Rep. 2023 Jul 3;13:10720. doi: 10.1038/s41598-023-37764-z (PMC10318030; doi:10.1038/s41598-023-37764-z)
Supplement: Supplementary file 3 — Supplementary Information 3. [file 41598_2023_37764_MOESM3_ESM.pdf]

Supplementary figure 3

Mut. Sig. relative contribution across bootstrapping (N = 100)

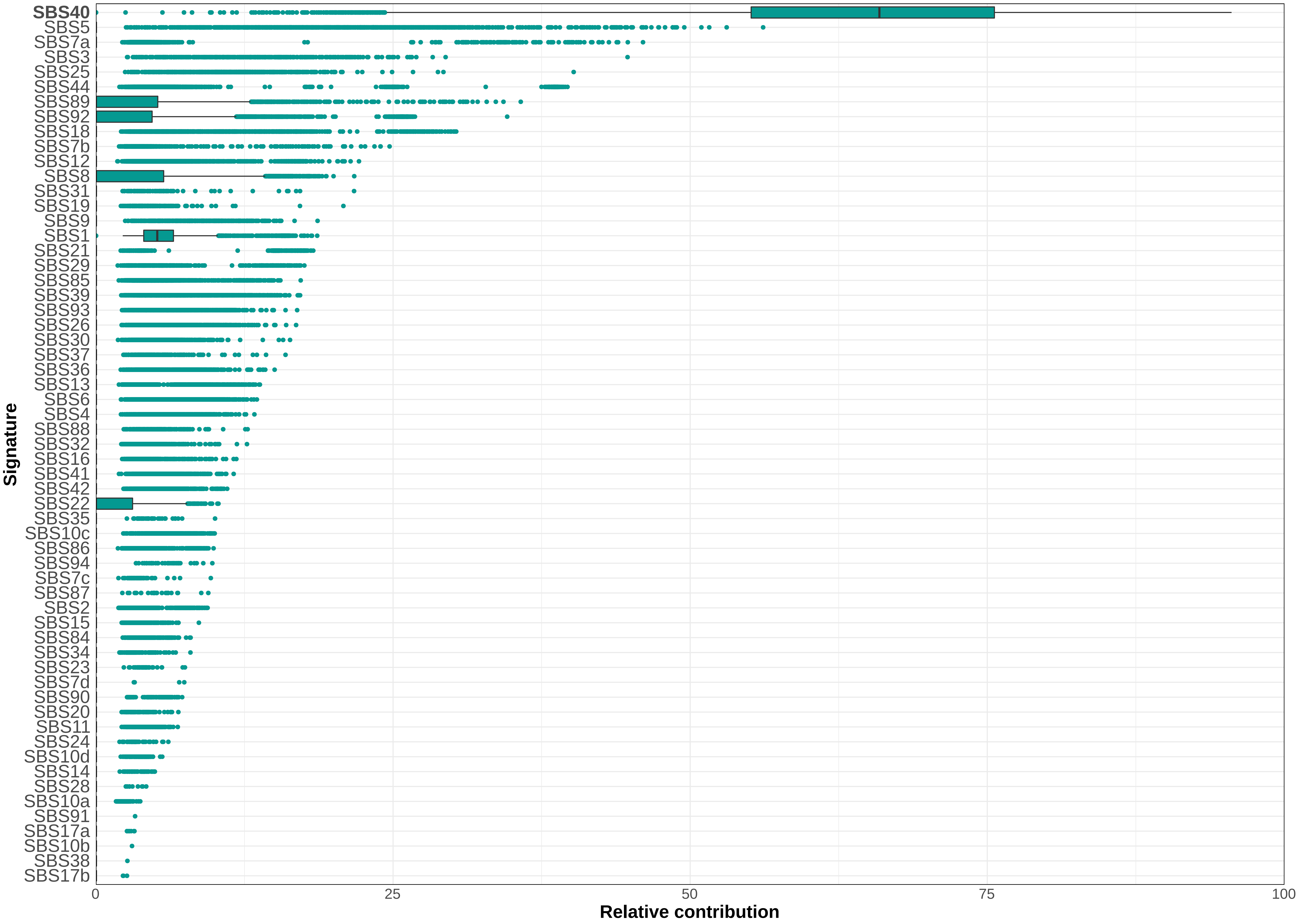

SBS signature 40 relative contribution at sample level across bootstrapping (N = 100)

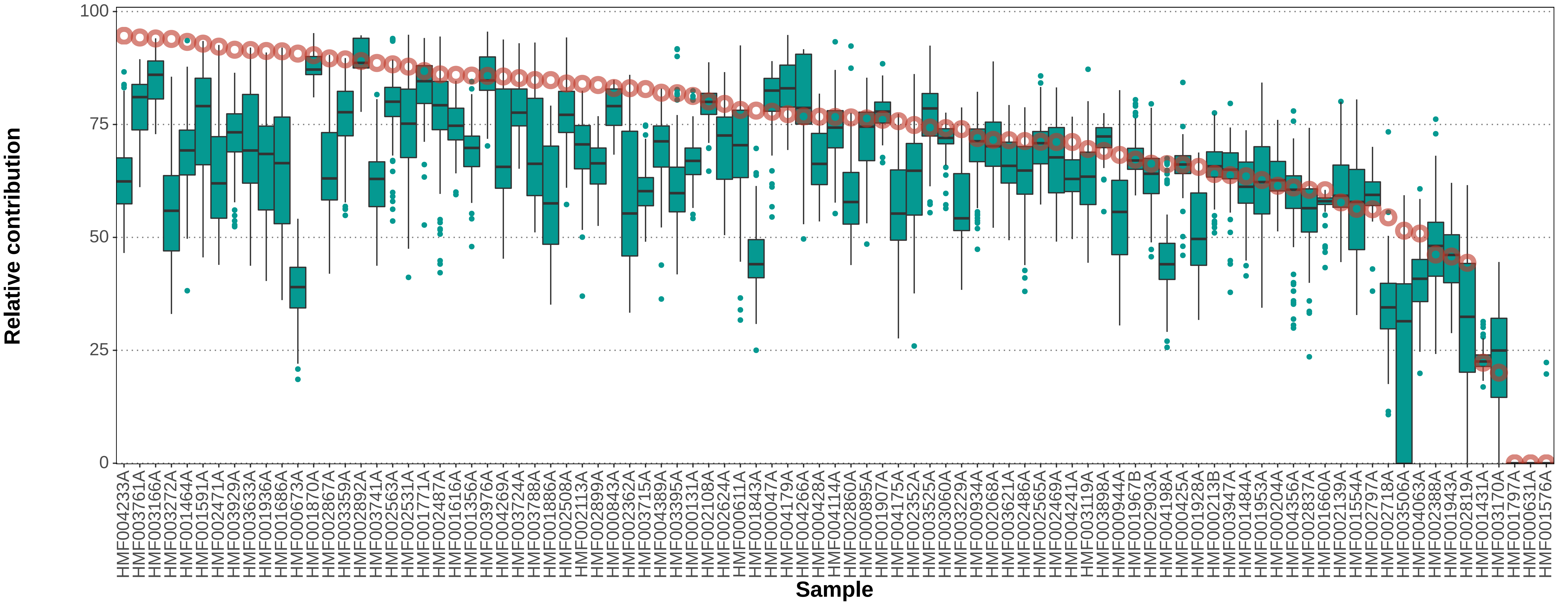

Supplementary figure 3: Relative contribution for COSMIC single base substitution mutational signatures across 100 bootstraps
Panel **A** X-axis shows the relative contribution of the bootstrapped mutational signatures and the y-axis shows the SBS signature ordered by maximum relative contribution assigned across 100 bootstraps. Panel **B** X-axis shows all samples and the y-axis shows the relative contribution of SBS40 assigned across 100 bootstraps, with the red circle illustrating the originally assigned relative contribution, for comparison.
