# Supplementary material for: The genomic and transcriptomic landscape of advanced renal cell cancer for individualized treatment strategies
Source: Sci Rep. 2023 Jul 3;13:10720. doi: 10.1038/s41598-023-37764-z (PMC10318030; doi:10.1038/s41598-023-37764-z)
Supplement: Supplementary file 4 — Supplementary Information 4. [file 41598_2023_37764_MOESM4_ESM.pdf]

Supplementary figure 4

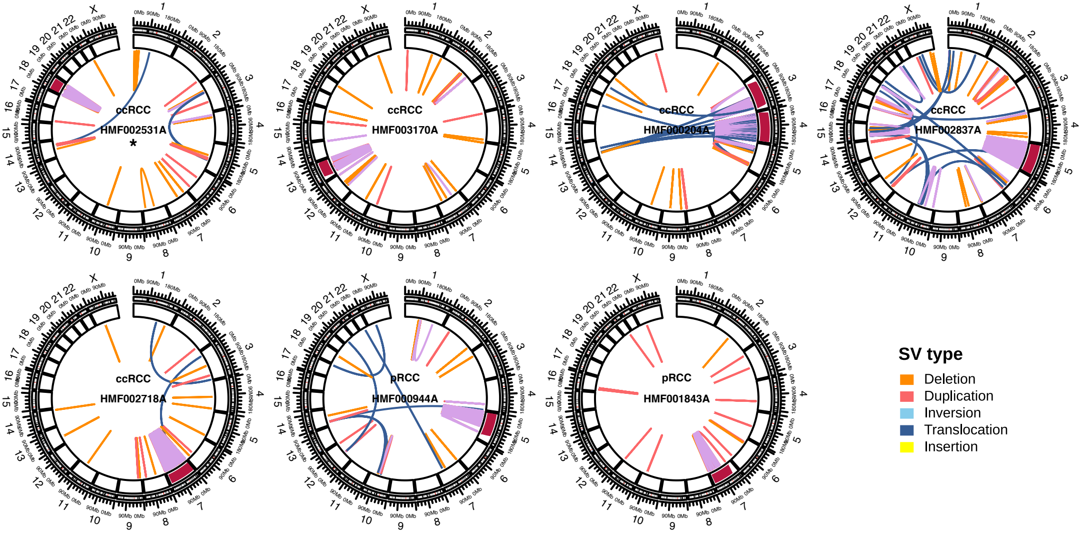

Supplementary figure 4: Circos plots of chromothripsis samples from the Renal Cell Carcinoma WGS sequencing cohort
Chromosomes indicated with a red block were flagged by chromothripsis detection. The star indicates the presence of a translocation from chromosome 3 to chromosome 5. Lines in the center indicate structural variants, with colors indicative of the type. ccRCC = clear cell renal cell carcinoma. pRCC = papillary renal cell carcinoma. Undefined subtype = renal cell carcinoma, with undefined subtype. chRCC = chromophobe
