# Supplementary material for: The genomic and transcriptomic landscape of advanced renal cell cancer for individualized treatment strategies
Source: Sci Rep. 2023 Jul 3;13:10720. doi: 10.1038/s41598-023-37764-z (PMC10318030; doi:10.1038/s41598-023-37764-z)
Supplement: Supplementary file 5 — Supplementary Information 5. [file 41598_2023_37764_MOESM5_ESM.pdf]

Supplementary figure 5

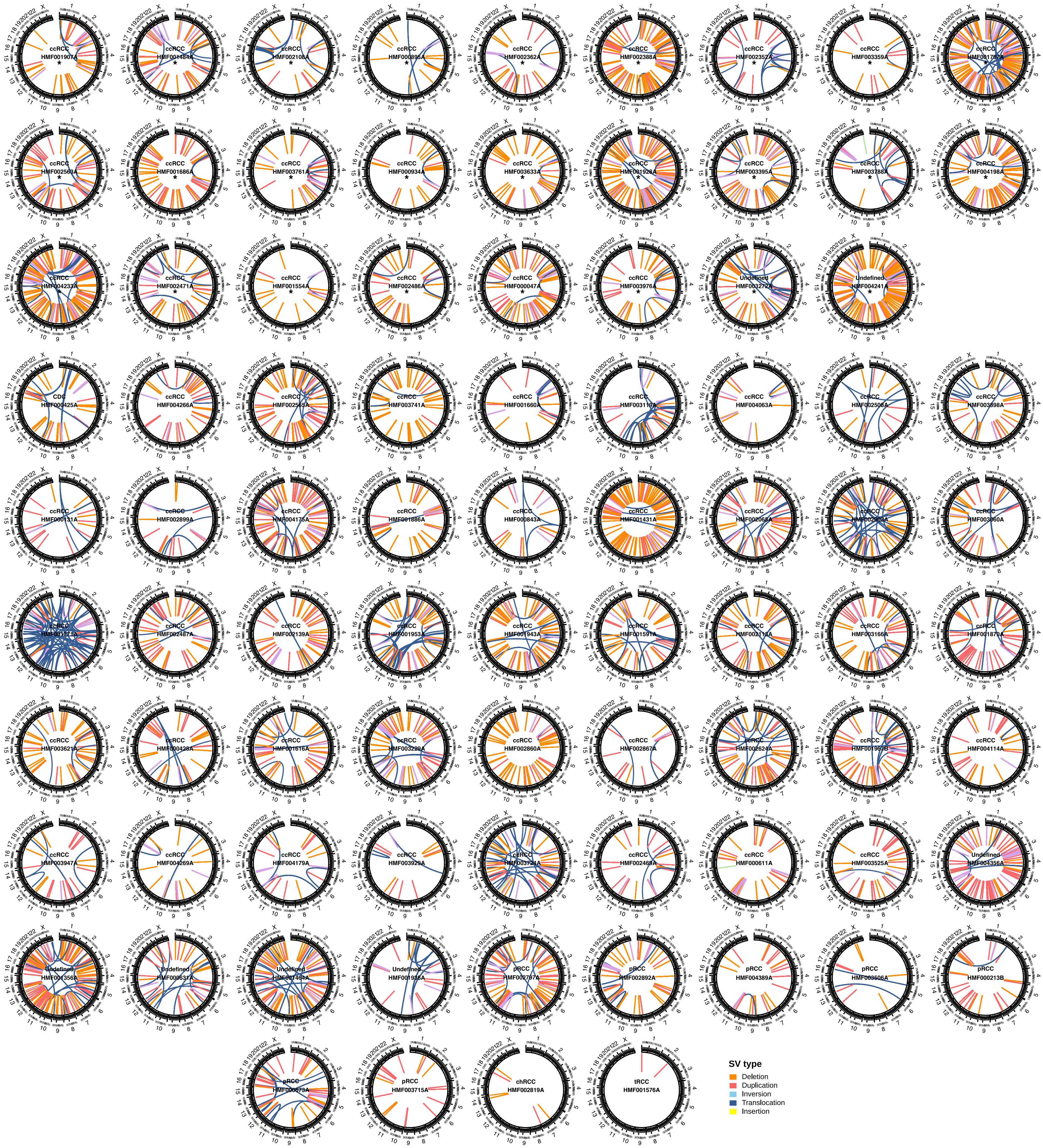

Supplementary figure 5: Circos plots of non-chromothripsis samples from the Renal Cell Carcinoma WGS sequencing cohort
Chromosomes indicated with a red block were flagged by chromothripsis detection. The stars indicates the presence of a translocation from chromosome 3 to chromosome 5. Lines in the center indicate structural variants, with colors indicative of the type. ccRCC = clear cell renal cell carcinoma. pRCC = papillary renal cell carcinoma. Undefined subtype = renal cell carcinoma, with undefined subtype. chRCC = chromophobe renal cell carcinoma. CDC = collecting duct carcinoma. tRCC = tubulocystic renal cell carcinoma.
