# Supplementary material for: The genomic and transcriptomic landscape of advanced renal cell cancer for individualized treatment strategies
Source: Sci Rep. 2023 Jul 3;13:10720. doi: 10.1038/s41598-023-37764-z (PMC10318030; doi:10.1038/s41598-023-37764-z)
Supplement: Supplementary file 6 — Supplementary Information 6. [file 41598_2023_37764_MOESM6_ESM.pdf]

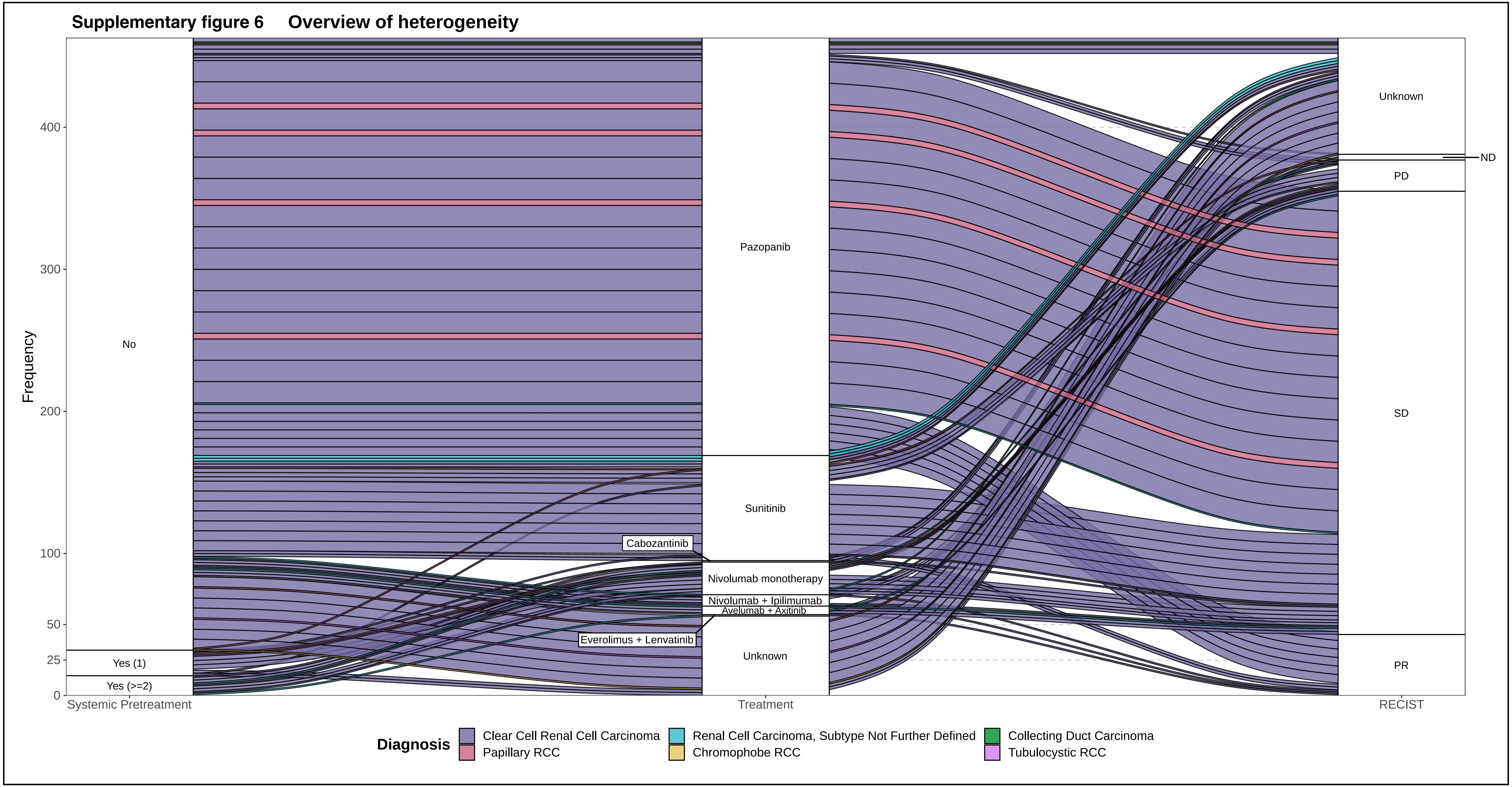

Supplementary figure 6: Clinical heterogeneity in Renal Cell Carcinoma WGS sequencing cohort
Alluvial diagram from pretreatment status (systemic treatment before biopsy) to treatment (therapies received after biopsy) and on to RECIST score (v1.1 after first treatment). Colors indicative of RCC subtype.
