# Supplementary material for: The genomic and transcriptomic landscape of advanced renal cell cancer for individualized treatment strategies
Source: Sci Rep. 2023 Jul 3;13:10720. doi: 10.1038/s41598-023-37764-z (PMC10318030; doi:10.1038/s41598-023-37764-z)
Supplement: Supplementary file 7 — Supplementary Information 7. [file 41598_2023_37764_MOESM7_ESM.pdf]

Supplementary figure 7

GISTIC2.0 analyses - Clear Cell Renal Cell Carcinoma

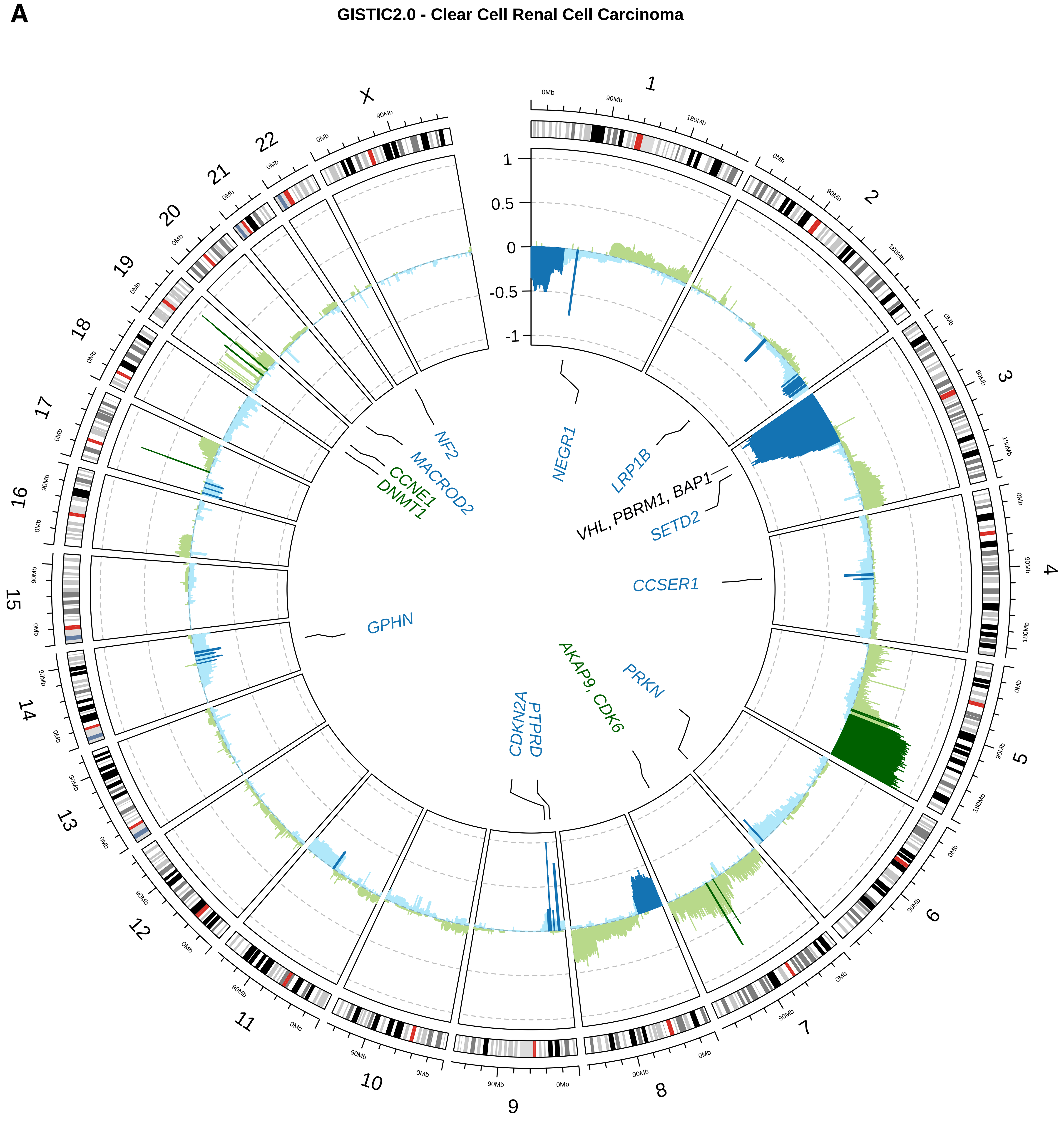

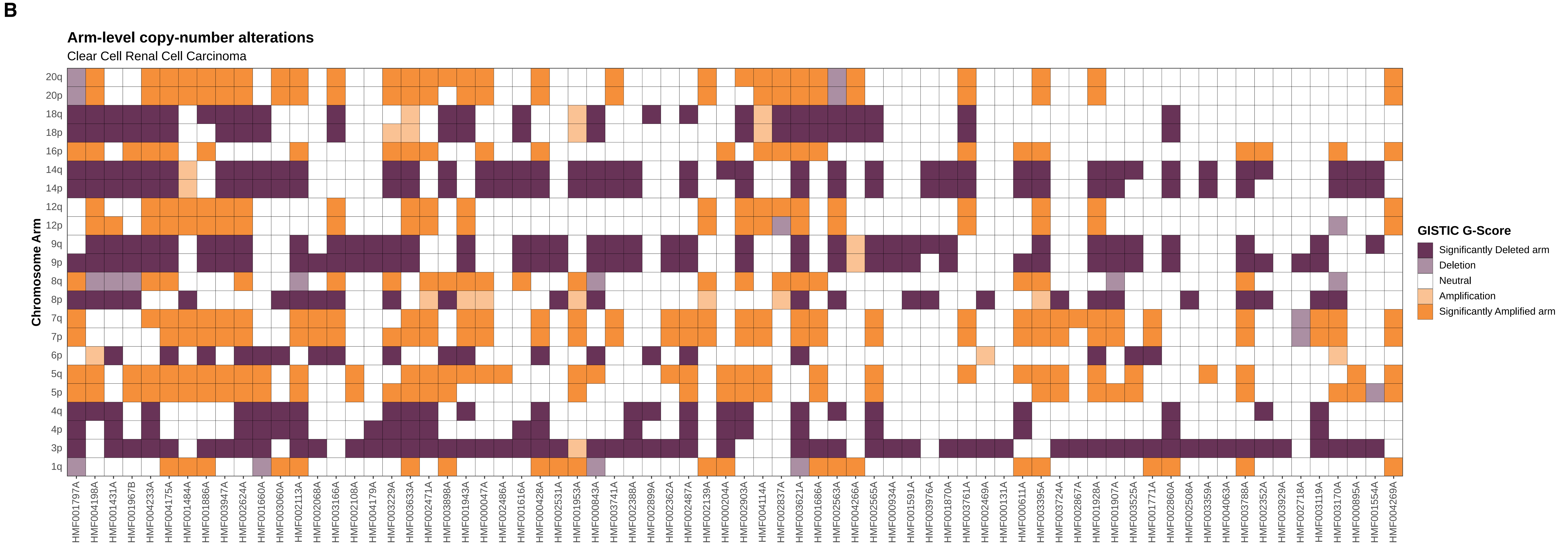

Supplementary Figure 7: Copy-number analysis in clear cell Renal Cell carcinoma (ccRCC)

Panel **A** is a circular overview of the copy-number alterations in the WGS advanced ccRCC cohort (N = 72). The outer ring shows the chromosomal ideogram, followed by a cohort-wide GISTIC2.0 G-score track with large peaks rounded to 1 and -1. Negative copy-numbers on the y-axis (blue) indicate deletions, with positive (green) indicating amplifications. The darker color is indicative of passing the statistical q-value threshold of 0.05. Known cancer driver genes overlapping copy-number peaks found to be significant by GISTIC2.0 are labelled in the center of the circle, utilizing the same color scheme as the G-score track. Panel **B** displays the arm-level copy-number alterations of significantly altered (q-value < 0.05) chromosome arms according to GISTIC2.0 (>50% of arm affected) in the WGS cohort (N = 72). Each column represents an ccRCC sample (ordered descendingly by tumor mutational burden), with the chromosome arm listed on the y-axis. Increase in copy-number is displayed in the grid cells in yellow and decrease in purple, white squares show no change in copy-number at arm-level.
