# Supplementary material for: The genomic and transcriptomic landscape of advanced renal cell cancer for individualized treatment strategies
Source: Sci Rep. 2023 Jul 3;13:10720. doi: 10.1038/s41598-023-37764-z (PMC10318030; doi:10.1038/s41598-023-37764-z)
Supplement: Supplementary file 8 — Supplementary Information 8. [file 41598_2023_37764_MOESM8_ESM.pdf]

Supplementary figure 8

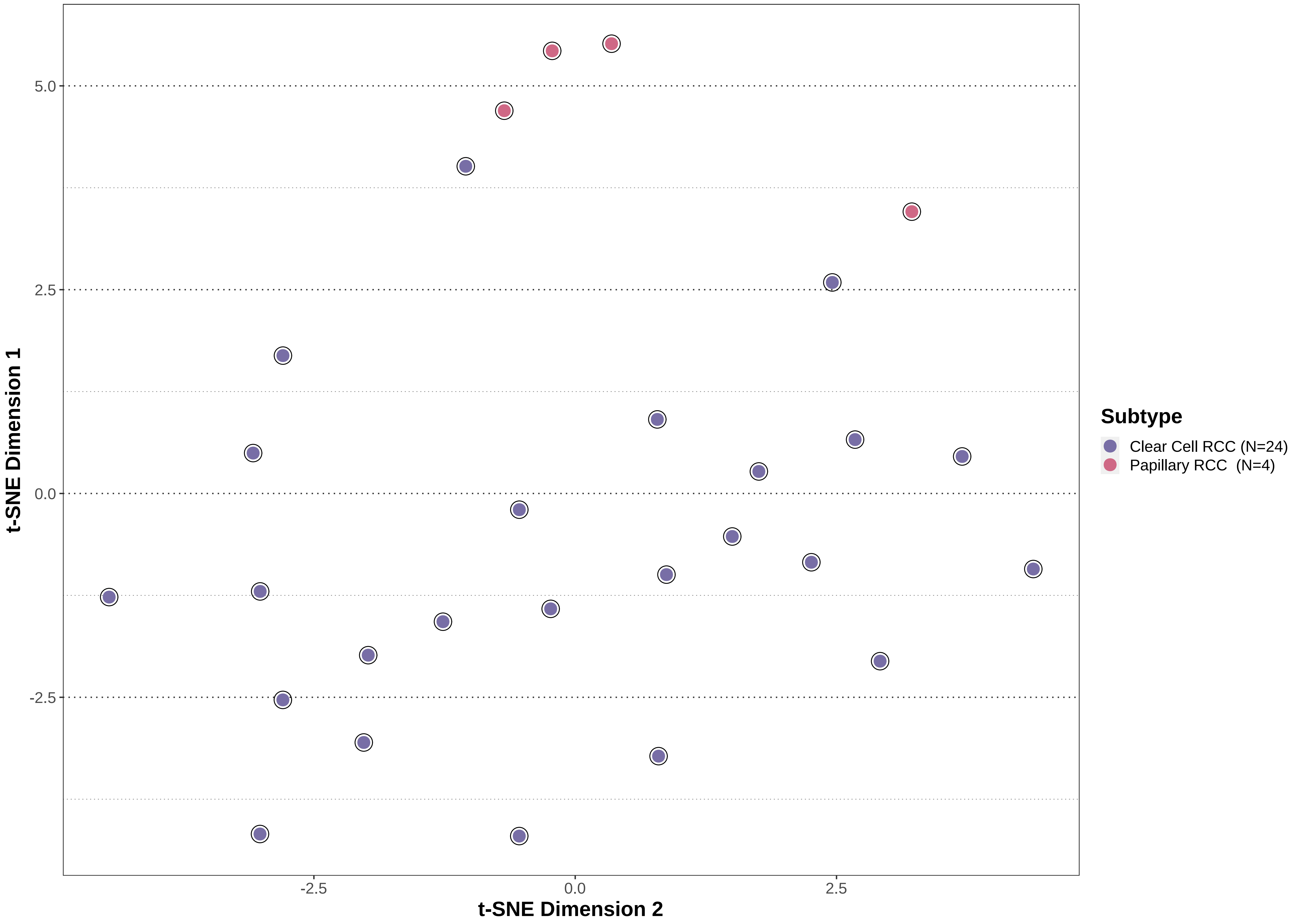

Supplementary figure 8: t-distributed stochastic neighbor (t-SNE) embedding plot of the expression analysis from RNA sequencing samples from cohort of clear cell Renal Cell Carcinoma (ccRCC; N = 24) and papillary Renal Cell Carcinoma (pRCC; N = 4)
CcRCC displayed in purple, pRCC in pink.
