# Supplementary material for: The genomic and transcriptomic landscape of advanced renal cell cancer for individualized treatment strategies
Source: Sci Rep. 2023 Jul 3;13:10720. doi: 10.1038/s41598-023-37764-z (PMC10318030; doi:10.1038/s41598-023-37764-z)
Supplement: Supplementary file 10 — Supplementary Information 10. [file 41598_2023_37764_MOESM10_ESM.docx]

**Supplementary data file – Full legend**

**Sheet ‘Fig2_characteristics’**

**Underlying data for figure 2 with small variants, structural variants and clinical factors**

| hmfSampleId | Hartwig Medical Foundation sample identifier |
| --- | --- |
| Genome.TMB | Genome-wide tumor mutational burden |
| tmbStatus | Tumor mutational burden grouped into bins |
| diagnosis | Renal Cell Carcinoma pathological subtype |
| totalSV | Total number of structural variants |
| value_Deletions | Absolute number of deletions |
| value_Tandem Duplications | Absolute number of tandem duplications |
| value_Inversions | Absolute number of inversions |
| value_Break-ends | Absolute number of break-ends |
| value_Translocations | Absolute number of translocations |
| value_Insertions | Absolute number of insertions |
| relSV_Deletions | Relative fraction of deletions |
| relSV_Tandem Duplications | Relative fraction of tandem duplications |
| relSV_Inversions | Relative fraction of inversions |
| relSV_Break-ends | Relative fraction of break-ends |
| relSV_Translocations | Relative fraction of translocations |
| relSV_Insertions | Relative fraction of insertions |
| genomePloidy | What is the whole-genome sequencing estimated ploidy? |
| chromothripsis | Was chromothripsis detected in this sample? TRUE/FALSE |
| C>A | Fraction of SNVs that change C>A |
| T>C | Fraction of SNVs that change T>C |
| C>G | Fraction of SNVs that change C>G |
| C>T | Fraction of SNVs that change C>T |
| T>A | Fraction of SNVs that change T>A |
| T>G | Fraction of SNVs that change T>G |
| hasSystemicPreTreatment | Did this patient receive systemic treatment prior to biopsy? Yes/No |
| treatmentNaive | Was this patient treatment naive at moment of biopsy? Yes/No |
| priestley | Has this patient been previously described in the referenced Priestley *et al.* paper? TRUE/FALSE |

**Sheet ‘Fig2_mutsigs’**

**Underlying data for figure 2 with mutational signature calling**

| hmfSampleId | Hartwig Medical Foundation sample identifier |
| --- | --- |
| diagnosis | Renal Cell Carcinoma pathological subtype |
| SBS1:SBS94 | Relative contribution of the respective SBS mutational signature |

**Sheet ‘Fig3_significant’**

**Underlying data for figure 3, specifically for the statistically significant driver genes, with small mutations, copy-number alterations and structural variants**

| hmfSampleId | Hartwig Medical Foundation sample identifier |
| --- | --- |
| SYMBOL | Gene symbol |
| Consequence.CNA | Type of detected copy-number alteration |
| Consequence.Mut | Type of detected (most deleterious) variant |
| Consequence.HGVSp | Effect on protein level of the (most deleterious) variant |
| Consequence.SV | Was a structural variant detected? Structural Variant/NA |
| diagnosis | Renal Cell Carcinoma pathological subtype |
| GISTIC2Peak | Is this gene part of a copy-number alteration peak? Peak ID/NA |
| dNdS | Has dNdS called this gene as significant within the RCC subtype? Significant/NA |

**Sheet ‘Fig3_selectedgenes’**

**Underlying data for figure 3, specifically for the selected genes, with small mutations, known germline mutations, copy-number alterations and structural variants**

| hmfSampleId | Hartwig Medical Foundation sample identifier |
| --- | --- |
| SYMBOL | Gene symbol |
| Consequence.CNA | Type of detected copy-number alteration |
| Consequence.Mut | Type of detected (most deleterious) variant |
| Consequence.HGVSp | Effect on protein level of the (most deleterious) variant |
| Consequence.SV | Was a structural variant detected? Structural Variant/NA |
| diagnosis | Renal Cell Carcinoma pathological subtype |
| germline | Type of variant detected in a gene known to be related to RCC on germline |

**Sheet ‘Fig4_iClusion’**

**Underlying data for figure 4 based on potentially actionable targets with the iClusion data**

| hmfSampleId | Hartwig Medical Foundation sample identifier |
| --- | --- |
| Gene | Gene symbol |
| Treatment | Treatment associated with potentially actionable target |
| Event | Event that is a potentially actionable target |
| EventMatch | Type of actionable target match, specific for the exact change or a gene-level match |
| Label | If the treatment is on-label or off-label for this pathological subtype assigned as described in the methods section. |
| subtype | Renal Cell Carcinoma pathological subtype |

**Sheet ‘Fig5_filtered.results’**

**Underlying data for figure 5 based on differential expression analysis, filtered for statistical significance**

| gene_name | Gene symbol |
| --- | --- |
| ENSEMBL | Ensembl gene identifier |
| baseMean | Average of normalized count values, dividing by size factors, taken over all samples |
| log2FoldChange | Fold change (log_2_) of differential expression analysis between Clear Cell Renal Cell Carcinoma and Papillary Renal Cell Carcinoma |
| lfcSE | Log fold change standard error |
| stat | Wald test statistic |
| pvalue | P-value |
| padj | Adjusted P-value (Benjamini-Hochberg corrected) |

**Sheet ‘Fig5_fgsea.results’**

**Underlying data for figure 5 based on the Gene Set Enrichment Analysis of statistically significant differentially expressed genes**

| pathway | Pathway name |
| --- | --- |
| pval | P-value |
| padj | Adjusted P-value (Benjamini-Hochberg corrected) |
| log2err | Expected error for the standard deviation of the P-value logarithm |
| ES | Enrichment score |
| NES | Normalized Enrichment Score, normalized to the mean enrichment of random samples of the same size |
| size | Number of genes involved in the pathway |

**Sheet ‘GISTIC_armlevel’**

**Data from the GISTIC2.0 arm-level analysis**

| hmfSampleId | Hartwig Medical Foundation sample identifier |
| --- | --- |
| Chromosome Arm | p/q arm identifier |
| value | GISTIC2.0 arm-level copy-number |
| diagnosis | Renal Cell Carcinoma pathological subtype |
| significant_Arm | Was the arm significantly affected by copy-number alterations according to GISTIC2.0? Yes/No |

**Sheet ‘GISTIC2_peaks’**

**Data from the GISTIC2.0 narrow peak analysis, filtered for statistical significance**

| seqnames | Chromosome name |
| --- | --- |
| start | Start position on chromosome of GISTIC2.0 peak |
| end | End position on chromosome of GISTIC2.0 peak |
| width | With of GISTIC2.0 peak |
| Unique.Name | Peak identifier |
| Descriptor | Peak descriptor based on cytoband |
| Wide.Peak.Limits | Peak limits (broad) |
| Peak.Limits | Peak limits (narrow) |
| Region.Limits | Copy-number region limits |
| q.values | Q-value of GISTIC2.0 statistical test |
| Residual.q.values.after.removing.segments.shared.with.higher.peaks | Q-value of GISTIC2.0 statistical test after removing segments shared with higher peaks |
| Amplitude.Threshold | Amplitude thresholds for this peak |
| overlapGenes.Final | Gene symbols over genes overlapping the GISTIC2.0 peak, based on driver genes (when present) |

**Sheet ‘Fusions’**

**Data from the fusion gene analysis, filtered for fusion pairs described in ChimerDB**

| hmfSampleId | Hartwig Medical Foundation sample identifier |
| --- | --- |
| SampleId | **THIS SHOULD BE DELETED BEFORE PUBLICATION** |
| ClusterId | Cluster identifier from fusion calling |
| Reportable | Should the fusion be reported in Hartwig Medical Foundation report? TRUE/FALSE |
| GeneNameUp | Gene symbol for gene upstream in fusion pair |
| TranscriptUp | Ensembl transcript identifier for gene upstream in fusion pair |
| ChrUp | Chromosome number for gene upstream in fusion pair |
| PosUp | Chromosomal position for gene upstream in fusion pair |
| StrandUp | Strand of gene upstream in fusion pair |
| RegionTypeUp | Was the fusion exonic or intronic for gene upstream in fusion pair? |
| FusedExonUp | Starting from which exon was the gene fused for gene upstream in fusion pair? |
| GeneNameDown | Gene symbol for gene downstream in fusion pair |
| TranscriptDown | Ensembl transcript identifier for gene downstream in fusion pair |
| ChrDown | Chromosome number for gene downstream in fusion pair |
| PosDown | Chromosomal position for gene downstream in fusion pair |
| StrandDown | Strand of gene downstream in fusion pair |
| RegionTypeDown | Was the fusion exonic or intronic for gene downstream in fusion pair? |
| FusedExonDown | Starting from which exon was the gene fused for gene downstream in fusion pair? |
| fusionPartners | Fusion name |
| knownFusion | Was this fusion present in ChimerDB? TRUE/FALSE |
| diagnosis | Renal Cell Carcinoma pathological subtype |

**Sheet ‘StructuralVariants’**

**Structural variants present in the cohort**

| hmfSampleId | Hartwig Medical Foundation sample identifier |
| --- | --- |
| seqnames | Chromosome name |
| start | Start position on chromosome for structural variant |
| end | End position on chromosome for structural variant |
| REF | Reference base at start position |
| ALT | Alternative base(s) and position of other breakpoint in structural variant |
| SVTYPE | Type of structural variant |
| diagnosis | Renal Cell Carcinoma pathological subtype |

**Sheet ‘chromothripsis’**

**Data from the chromothripsis analysis, filtered on statistical significance**

| hmfSampleId | Hartwig Medical Foundation sample identifier |
| --- | --- |
| diagnosis | Renal Cell Carcinoma pathological subtype |
| chrom | Chromosome chromothripsis event was detected on |
| start | Start position of chromothripsis event |
| end | End position of chromothripsis event |
| number_DEL | Number of deletions involved in chromothripsis event |
| number_DUP | Number of duplications involved in chromothripsis event |
| number_h2hINV | Number of head-to-head inversions involved in chromothripsis event |
| number_t2tINV | Number of tail-to-tail inversions involved in chromothripsis event |
| number_TRA | Number of translocations involved in chromothripsis event |
| clusterSize_including_TRA | Size of the chromothripsis cluster including translocations |
| number_SVs_sample | Number of structural variants in sample |
| number_CNV_segments | Number of copy-number alteration segments in sample |
| pval_fragment_joins | P-value of statistical test for even distribution for the different types of structural variants |
| chr_breakpoint_enrichment | P-value of statistical test to determine chromothripsis breakpoints in epigenomic marks |
| pval_exp_chr | P-value of exponential distribution of breakpoints test for the whole chromosome |
| pval_exp_cluster | P-value of exponential distribution of breakpoints test for just the cluster |
| max_number_oscillating_CN_segments_2_states | Maximum number of oscillating copy-number segments on just two states in the cluster |
| max_number_oscillating_CN_segments_3_states | Maximum number of oscillating copy-number segments on three states |
| number_CN_segments_chr | Number of copy-number segments on the whole chromosome |
| max_number_oscillating_CN_segments_2_states_chr | Maximum number of oscillating copy-number segments on just two states on the whole chromosome |
| max_number_oscillating_CN_segments_3_states_chr | Maximum number of oscillating copy-number segments on three states on the whole chromosome |
| inter_number_DEL | Number of interchromosomal deletions |
| inter_number_h2hINV | Number of interchromosomal head-to-head inversions |
| inter_number_t2tINV | Number of interchromosomal tail-to-tail inversions |
| inter_number_DUP | Number of interchromosomal duplications |
| inter_pval_fragment_joins | P-value of statistical test for even distribution for the different types of interchromosomal structural variants |
| inter_other_chroms | Other chromosome affected by structural variants overlapping the chromothripsis region chromosome |
| inter_other_chroms_coords_all | Coordinates on other chromosome affected by structural variants overlapping the chromothripsis region chromosome |
