# Supplementary material for: The genomic and transcriptomic landscape of advanced renal cell cancer for individualized treatment strategies
Source: Sci Rep. 2023 Jul 3;13:10720. doi: 10.1038/s41598-023-37764-z (PMC10318030; doi:10.1038/s41598-023-37764-z)
Supplement: Supplementary file 9 — Supplementary Information 9. [file 41598_2023_37764_MOESM9_ESM.pdf]

Supplementary figure 9

A

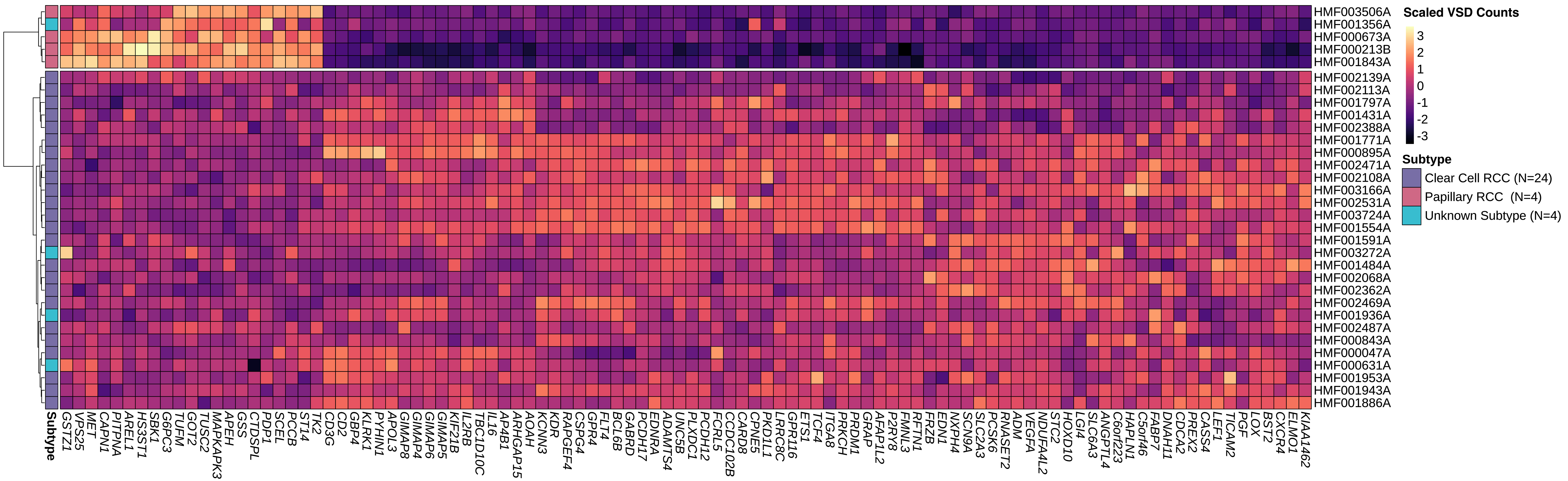

B

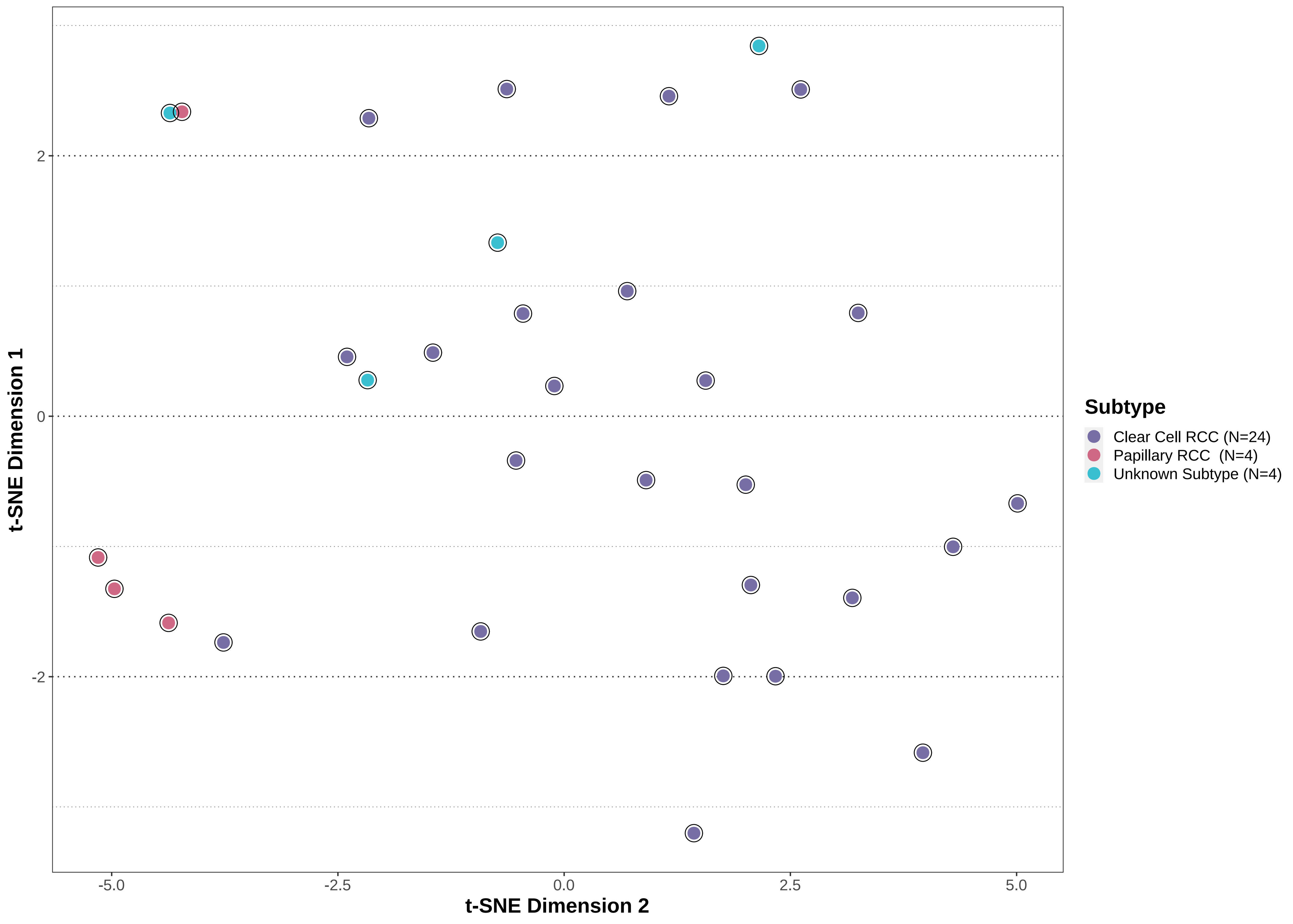

Supplementary figure 9: Heatmap (A) and t-distributed stochastic neighbor embedding (t-SNE) plot (B) of samples from the Renal Cell Carcinoma RNA sequencing cohort

Illustrative figures to visualize clustering of RCC samples with undefined subtype included. Gene selection and order in **A** is based on variance stabilized values with unsupervised clustering of Z-scores of the top 100 genes (based on smallest adjusted p-value) statistically significant genes, based on the differential expression analysis between ccRCC (N = 24) and pRCC (N = 4). **B** t-SNE plot of variance stabilized read counts of all protein coding genes in ccRCC, pRCC and RCC with undefined subtype.
